# Assessment of the Mode of Action Underlying the Effects of GenX in Mouse Liver and Implications for Assessing Human Health Risks

**DOI:** 10.1177/0192623320905803

**Published:** 2020-03-06

**Authors:** Grace A. Chappell, Chad M. Thompson, Jeffrey C. Wolf, John M. Cullen, James E. Klaunig, Laurie C. Haws

**Affiliations:** 1 ToxStrategies, Inc, Asheville, NC, USA; 2 ToxStrategies, Inc, Katy, TX, USA; 3 Experimental Pathology Laboratories, Sterling, VA, USA; 4 North Carolina State University College of Veterinary Medicine, Raleigh, NC, USA; 5 Indiana University, School of Public Health, Bloomington, IN, USA; 6 ToxStrategies, Inc, Austin, TX, USA

**Keywords:** transcriptomics, perfluoroalkyl and polyfluoroalkyl substances (PFAS), GenX, mode of action, single-cell necrosis, peroxisome proliferator-activated receptor α (PPARα)

## Abstract

GenX is an alternative to environmentally persistent long-chain perfluoroalkyl and polyfluoroalkyl substances. Mice exposed to GenX exhibit liver hypertrophy, elevated peroxisomal enzyme activity, and other apical endpoints consistent with peroxisome proliferators. To investigate the potential role of peroxisome proliferator-activated receptor alpha (PPARα) activation in mice, and other molecular signals potentially related to observed liver changes, RNA sequencing was conducted on paraffin-embedded liver sections from a 90-day subchronic toxicity study of GenX conducted in mice. Differentially expressed genes were identified for each treatment group, and gene set enrichment analysis was conducted using gene sets that represent biological processes and known canonical pathways. Peroxisome signaling and fatty acid metabolism were among the most significantly enriched gene sets in both sexes at 0.5 and 5 mg/kg GenX; no pathways were enriched at 0.1 mg/kg. Gene sets specific to the PPARα subtype were significantly enriched. These findings were phenotypically anchored to histopathological changes in the same tissue blocks: hypertrophy, mitoses, and apoptosis. In vitro PPARα transactivation assays indicated that GenX activates mouse PPARα. These results indicate that the liver changes observed in GenX-treated mice occur via a mode of action (MOA) involving PPARα, an important finding for human health risk assessment as this MOA has limited relevance to humans.

## Introduction

Perfluoroalkyl and polyfluoroalkyl substances (PFAS) are anthropogenic compounds used in a variety of industrial and consumer products, including industrial surfactants and emulsifiers, firefighting foams, stain-resistant coatings for textiles, oil-resistant coatings for food packaging, personal care products, and nonstick coatings on cookware.^1-3^ The same physical properties that make PFAS useful in the aforementioned applications also make them resistant to biodegradation, hydrolysis, and photooxidation, resulting in their persistence in the environment.^1^ Many PFAS have long biological half-lives and therefore remain detectible in blood samples from exposed humans for many years.^4^ Due to concerns about persistence, long-chain PFAS such as perfluorooctanoic acid (PFOA) and perfluorooctane sulfate (PFOS) have been phased out of production in favor of PFAS with more favorable characteristics, such as lower toxicity, decreased bioaccumulation, and potentially less environmental persistence.

Ammonium 2,3,3,3-tetrafluoro-2-(heptafluoropropoxy)-propanoate (CASRN 62037-80-3), also known as “GenX,” is an example of an alternative PFAS with limited evidence for bioaccumulation.^5^ Various groups have concluded that short- and long-term toxicity studies on GenX indicate that the liver is the primary target of toxicity following oral exposure.^6,7^ Several studies of GenX in the mouse have reported hepatocyte “single-cell necrosis” in the liver^8,9^ and some recent risk assessments for GenX have been based on concerns of liver necrosis due to the presence of the so-called “single-cell necrosis.”^7^ However, more recent diagnostic criteria recommendations indicate that the older and broader term “single-cell necrosis” should be replaced with a delineation between necrotic and apoptotic cell death.^10^ These forms of cell death can be distinguished by H&E staining and may provide better insight into the mode of action (MOA) of the chemical. We recently reevaluated liver sections from one such study (a reproductive toxicity study conducted in 2010^9^) that was used as the basis of a draft toxicity value proposed by the US Environmental Protection Agency^7^ and concluded that the single-cell necrosis was more consistent with apoptosis, using current standards and practices.^6^ In that reevaluation, mitosis was increased concomitantly with apoptosis, indicating a potential homeostatic response to increased cell proliferation.^6^ The increase in hepatic cell proliferation observed in that study is consistent with evidence for GenX activation of PPARα signaling, as evidenced by increased peroxisomal enzyme activity, liver hypertrophy, and transcriptomic signaling.^6,11^ Relatedly, other PFAS have been shown to activate PPARα.^12^

To further explore potential mechanisms of toxicity and relevance of such underlying the liver lesions observed in mice, herein we report transcriptomic and immunohistochemical analyses in male and female mice from a 90-day Organisation for Economic Co-operation and Development (OECD) 408 guideline oral toxicity study.^8^ In addition, similar to the reevaluation of livers from male and female mice from a reproductive toxicity study^9^ that was conducted in our earlier study,^6^ we reevaluated livers from the 90-day OECD 408 guideline oral toxicity study^8^ using current histopathological criteria to allow for phenotypic anchoring to findings from transcriptomic analyses of liver tissues from this same study. Transcriptomic data can provide additional and/or supporting information regarding mechanisms of disease associated with specific exposure scenarios.^13-17^ The transcriptomic signatures in male and female mice were also anchored to phenotypes determined by H&E staining of sections from the same tissue blocks. Gene set enrichment analysis and dose–response modeling were also conducted to understand alterations in biological and disease processes across treatment groups and to contribute to a better understanding of the MOA for GenX. Moreover, H&E-stained sections from the original 90-day OECD 408 guideline study were reevaluated using the most recent pathology diagnostic criteria and compared to results from caspase-3 immunostaining. The overall weight of the evidence was then considered collectively to inform the MOA underlying the liver effects observed in mice. This information is important for understanding the relevance of the findings observed in mice in assessing human health risks.

## Materials and Methods

### Animal Husbandry and Exposure Conditions

The subchronic toxicity of ammonium 2,3,3,3-tetrafluro-2-(heptafluoropropoxy)-propanoate (CASRN 62037-80-3, molecular weight 347.08, tradename GenX) was evaluated in a 90-day oral gavage study in male and female Crl: CD1(ICR) mice (n = 10 per sex per concentration of GenX), as reported by MacKenzie.^8^ The test substance had a purity of 84%. The mice were dosed with the test substance at 0.1, 0.5, or 5 mg/kg body weight (bw)/day (d), with a control group dosed with deionized water. The study was conducted at E.I. du Pont de Nemours and Company, DuPont Haskell Global Centers for Health & Environmental Sciences (Delaware) in compliance with all applicable sections of the Final Rules of the Animal Welfare Act regulations (9CFR) and the Guide for the Care of Use of Laboratory Animals.^18^ The study complied with OECD Section 4 Part 408: Repeated Dose Oral Toxicity Study in Rodents, *Guidelines for Testing of Chemicals* (1998). Animals were housed individually at a temperature of 18°C to 26ºC and relative humidity of 30% to 70% on an approximate 12-hour light/dark cycle. Animals were provided tap water and PMI^®^ Nutrition International, LLC (Gray Summit, Missouri) Certified Rodent LabDiet^®^ 5002 ad libitum. Doses were formulated in deionized water and prepared weekly and verified analytically. Mice were euthanized by CO_2_ anesthesia and exsanguination. Livers were fixed in 10% neutral-buffered formalin, embedded in paraffin, and sections approximately 5 to 6 µm in thickness were mounted to slides for H&E staining.

### Histopathological Examination

#### Reevaluation of hepatocellular single-cell necrosis

The term “single-cell necrosis” previously represented multiple forms of hepatocellular death; however, more recent guidance recommends histologically distinguishing “single-cell necrosis” as apoptosis or necrosis because the distinctions can potentially provide insight into MOA.^10^ Therefore, H&E-stained liver sections from male and female mice exposed to GenX in the afore-mentioned 90-day OECD 408 guideline oral toxicity study^8^ were reevaluated by a board-certified veterinary pathologist (J.M.C.). In accordance with a clarification of the criteria recommended by Elmore et al,^10^ the 2 terms used to classify hepatocyte death were apoptosis and necrosis, using nomenclature from the Terminology Recommendations from the International Harmonization of Nomenclature and Diagnostic Criteria (INHAND) in Apoptosis/Necrosis Working Group. Additionally, the Societies of Toxicologic Pathology INHAND Nomenclature for Non-neoplastic Findings of the Rodent Liver was also consulted.^19^ Apoptosis, necrosis, and mitosis were scored by tallying the number of cells across 5 fields (×20 objective). Severity grades for apoptosis and necrosis were assigned as follows: grade 0 = no evident change, grade 1 = minimal (present in 1-5 hepatocytes/5 ×20 fields), grade 2 = mild, (present in 6-20 hepatocytes/5 ×20 fields), and grade 3 = moderate (present in 21-40 hepatocytes/5 ×20 fields).

#### Immunostaining for caspase-3

Liver specimens in paraffin blocks (2 portions of liver per block) from male and female mice from the same 90-day OECD 408 guideline toxicity study^8^ were received by Experimental Pathology Laboratories (EPL; Research Triangle Park, North Carolina). A single 4- to 6-µm section was microtomed from each block (1 block per concentration per sex), mounted on a glass slide, and stained for activated caspase-3 by immunohistochemistry.^20^ Primary antibody includes cleaved caspase-3, rabbit monoclonal (Cell Signaling (Danvers, Massachusetts), #9664); secondary polymer: MACH2 Rabbit HRP Polymer (Biocare Medical (Pacheco, California), RHRP520); chromagen: diaminobenzidine; counterstain: Richard-Allen Hematoxylin, 7231. Positive controls consisted of sections of thymus and intestine. Negative controls consisted of sections of thymus and intestine in which rabbit immunoglobulin G was substituted for the caspase-3 primary antibody. Stained slides were evaluated at EPL (Sterling, Virginia). The staining was scored using the following scale: grade 1 (minimal) = pale cytoplasmic labeling of Kupffer cells and occasional histiocytic macrophages; grade 2 (mild) = grade 1 attributes plus additional finely granular cytoplasmic labeling of low numbers of hepatocytes; grade 3 (moderate) = grade 2 attributes plus additional punctate labeling of hepatocytes, occasional apoptotic bodies, and rare hepatocyte nuclei; and grade 4 (marked) = grade 3 attributes plus more frequent punctate labeling of apoptotic bodies and granular cell debris within the cytoplasm of Kupffer cells and macrophages.

### RNA Sequencing

Using the same aforementioned formalin-fixed, paraffin-embedded liver samples from the 90-day OECD 408 guideline oral toxicity study,^8^ a single 4- to 6-µm section was microtomed from one block per animal for 5 animals per sex per concentration group and mounted on a glass slide (uncovered), yielding a total of 40 samples for RNA sequencing. The first 5 samples in sequential order (by animal number) from each treatment group were used for transcriptomic analyses. Slides were shipped to BioSpyder Technologies (Carlsbad, California) where the unstained liver sections were uniformly scraped from the slides and processed according to the TempO-Seq^®^ protocol, as previously described.^21^ DNA libraries from each liver sample from each animal were sequenced using a HiSeq 2500 Ultra-High-Throughput Sequencing System (Illumina, San Diego, California).

### Data Processing and Analysis

Sequencing data were analyzed using packages in the R software environment, version 3.5.2 (cran.r-project.org/). The number of sequenced reads per probe were extracted from FASTQ files generated from the sequencing experiment, with each probe representing a gene-specific sequence. The DESeq2 R package (v122.2)^22^ was used to normalize data such that sample-to-sample variation in sequencing depth was considered. Samples with an overall sequencing depth (total reads across all probes) lower than 2 standard deviations below the mean sequencing depth across all samples were excluded from the comparative analysis. Count data from all samples that passed this sequencing depth quality criterion were added to the DESeq experiment for normalization and for further comparative analyses.

#### Identification of genes with significant differential expression across concentrations

Statistical methods within DESeq2 were used to identify differentially expressed genes (DEGs) associated with exposure by conducting comparisons between groups that share a characteristic.^22^ In the present study, the various treatment groups were compared to controls of the same sex. Differentially expressed probes (DEPs) were defined as those with a false discovery rate (FDR) <10% for any chosen comparison between treatment groups, based on *P* values adjusted for multiple testing using the Benjamini and Hochberg (BH) procedure,^22^ paralleling methods previously used to analyze RNA sequencing data.^23,24^ Unique DEGs were identified from respective DEPs.

#### Identification of pathway-level alterations across concentrations

Biological pathways that were associated with the transcriptomic response profiles were identified by pathway enrichment analysis. For genes for which multiple probes were used to measure expression, the probe with the highest sequencing count across all samples was selected as the representative gene to be used in the pathway analyses. Mouse gene identifiers were converted into human identifiers, when available, using the R package biomaRt (v2.38.0) based on the Ensembl genome database (http://uswest.ensembl.org/index.html). Human gene identifiers were then queried for enrichment of gene sets within the canonical pathway (CP) subcollection available through the Molecular Signatures Database (MSigDB, http://software.broadinstitute.org/gsea/msigdb/index.jsp), which includes gene sets from several pathway databases (eg, the BioCarta online maps of metabolic and signaling pathways [BIOCARTA]),^25^ the Kyoto Encyclopedia of Genes and Genomes (KEGG),^26^ the Pathway Interaction Database (PID),^27^ and the Reactome database of reactions, pathways, and biological processes (REACTOME).^28^

Enrichment of gene sets and pathways was evaluated by two different methods: the first follows the analysis employed by the gene set enrichment analysis (GSEA) platform made available by the Broad Institute (http://software.broadinstitute.org/gsea/index.jsp), the second employed a more simple hypergeometric test. The GSEA method^29^ determines whether sets of genes (eg, the constituents of a molecular signaling pathway) are significantly concordant between various defined groups (in the case presented herein, different doses) based on a ranking metric (in this case, the statistical measure of significance of expression differences between treated and control mice). The GSEA statistical method was applied within the Platform for Integrative Analysis of Omics data (PIANO) R package (v1.22.0).^30^ Gene set enrichment significance was calculated using permutation-based nominal *P* values based on weighted Kolmogorov-Smirnov test enrichment scores and adjusted for multiple hypothesis testing by calculating FDRs using the BH method, as previously described.^29^ Gene sets with an FDR <10% were considered to be significantly enriched. For the hypergeometric test, all DEGs for each treatment group (ie, an FDR of <10% as described above) were tested for overrepresentation among the gene sets in the CP subcollection using the Fisher combined probability test function within the PIANO package. Gene sets with an FDR <10% were considered significantly enriched. Finally, to further supplement the enrichment analysis with the GSEA and hypergeometric tests using MSigDB collections, we analyzed the lists of significant DEGs for the 5 mg/kg bw dose groups using the Enrichr online tool (https://amp.pharm.mssm.edu/Enrichr/), which employs additional sources of gene set collections.^31^

#### Benchmark dose analysis

Dose–response modeling was conducted using the BMDExpress software (v2.2).^32^ Briefly, probe IDs from the TempO-Seq experiment were translated into mouse Ensembl IDs using the biomaRt R package (v2.38.0). Normalized expression data for all samples as generated using DESeq2 were then loaded into BMDExpress without transformation. A Williams trend test (with *P* value cutoff = .05) was used to identify genes altered by GenX exposure. No fold-change filters or correction for multiple tests were applied. Benchmark dose (BMD) analysis was conducted using the following models: linear, power, hill, 2° and 3° polynomial, and exponential models 2 to 5. The models were run assuming constant variance and a benchmark response (BMR) of 1 standard deviation. Functional classification was conducted using the gene set collections available within the BMDExpress software (Gene Ontology [GO] terms and Reactome gene sets), based on significantly dose-responsive genes (ie, all genes with BMD *P* values ≤.1), and removing genes according to the default parameters as follows: genes with BMD/BMDL >20, BMDU/BMDL 40, BMDs above 5 mg (highest dose), and/or genes with a BMD > 10-fold below the lowest positive dose. No filters for minimum or maximum number of genes per gene set were applied. Benchmark doses for the gene sets were also calculated. Additional settings for the BMD modeling and pathway/signaling analyses can be found in the Supplemental Materials.

### Data Availability

RNA sequencing data are publicly available at NCBI’s Gene Expression Omnibus^33^ (https://www.ncbi.nlm.nih.gov/geo/) (GEO series accession number GSE135943).

## Results

### Reevaluation of Hepatocellular Single-Cell Necrosis

MacKenzie^8^ previously reported that exposure to GenX for 90 days produced a dose-dependent increase in liver hypertrophy and liver organ weight in mice (Table 1). Similarly, a dose-dependent increase in “single-cell necrosis” was noted (Table 1). Single-cell necrosis was reevaluated using more current diagnostic criteria (as described by Elmore et al^10^). No necrotic cells were observed in male or female livers in any dose groups. In contrast, apoptosis was observed in 10 of 10 males and 1 of 10 females at 5 mg/kg/d (Table 1, Supplemental Table S1; see Figure S1 for representative image), with average severity scores of 1.9 and 0.1, respectively (Table 1). In male mice, the mitotic cells were observed only in the highest exposure group, with an average severity score of 0.7 (Table 1). In female mice, only 1 of 10 mice exhibited increased mitosis, which only occurred in the 0.5 mg/kg group (Table 1). Individual scores of necrosis, apoptosis, and mitosis in the liver are presented in Supplemental Table S1.

**Table 1.** Group Incidence and Mean Scores for Various Histopathological Metrics Evaluated in the Livers of Mice.

	Incidence as Diagnosed by MacKenzie^8^			Reevaluation of Slides From the MacKenzie’s study.^8^ Updated Diagnostic Criteria for Apoptosis as Described by Elmore^10^ Were Applied for the Determination of Apoptosis Versus Necrosis					IHC Staining of Liver Sections From MacKenzie^8^
GenX Treatment (mg/kg)	Liver Weight (g), mean (SD)	Hepatocellular Hypertrophy (Incidence)	Single-Cell Necrosis (Incidence)	Apoptosis (Incidence)	Mitosis (Incidence)	Necrosis (Incidence)	Apoptosis Score (Mean)	Mitosis Score (Mean)	Caspase-3 Staining Grade (Mean)
Males									
0	1.96 (0.27)	0/10	0/10	0/10	0/10	0/10	0	0	1
0.1	2.02 (0.17)	0/10	0/10	0/10	0/10	0/10	0	0	1
0.5	2.19 (0.27)	8/10	0/10	0/10	0/10	0/10	0	0	2
5	5.14 (1.81)	10/10	10/10	10/10	5/10	0/10	1.9	0.7	3.6
Females									
0	1.69 (0.39)	0/10	0/10	0/10	0/10	0/10	0	0	1
0.1	1.7 (0.14)	0/10	0/10	0/10	0/10	0/10	0	0	1
0.5	1.75 (0.30)	0/10	0/10	0/10	1/10	0/10	0	0.1	1.2
5	2.87 (0.99)	10/10	1/10	1/10	0/10	0/10	0.1	0	3

Abbreviations: IHC, immunohistochemistry; SD, standard deviation.

### Caspase-3 Immunostaining

**Figure 1.**
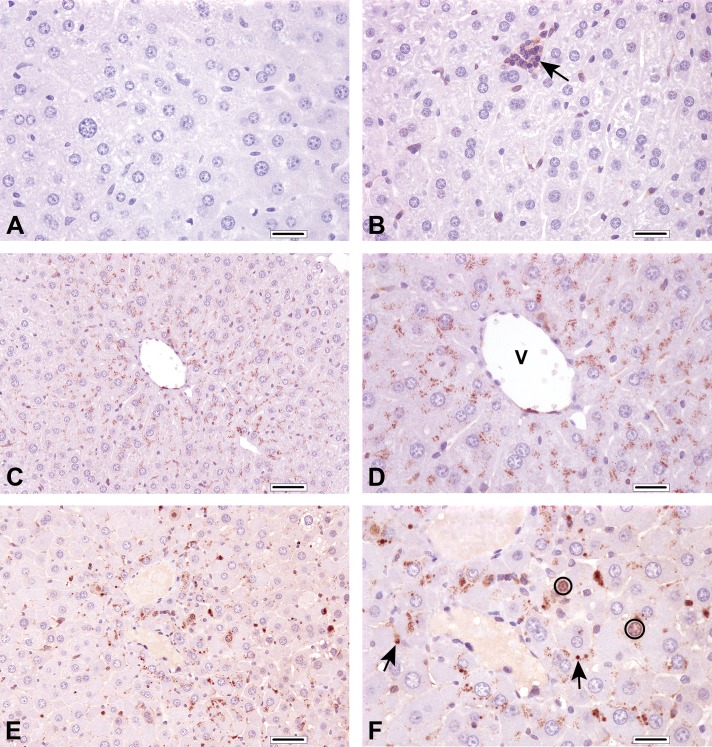
Caspase-3 staining. A, Liver section from untreated male mouse stained with primary antibody replaced by nonspecific rabbit immunoglobulin G. B, Liver section from untreated male mouse stained for caspase-3. Note the faint cytoplasmic immunolabeling in Kupffer cells and leukocyte aggregates (arrow). C and D, Liver section from male mouse exposed to 0.5 mg/kg GenX at original objective ×20 (C) and original objective ×40 (D) magnification. Note the fine stippling of cytoplasm in hepatocytes with normal morphology, as well as the more pronounced staining in hepatocytes surrounding the central vein (V). This section was scored as grade 2. E and F, Liver section from male mouse exposed to 5 mg/kg GenX at original objective ×20 (E) and original objective ×40 (F) magnification. Note immunolabeling of the hepatocyte cytoplasm, plus labeling of variably sized spherical to irregular apoptotic bodies (arrows) and, rarely, hepatocyte nuclei (circled). This section was scored as grade 4. A, B, D, and F, Bar = 40 μm. C and E, Bar = 50 μm.

Treatment with GenX for 90 days produced a dose-dependent increase in caspase-3 immunostaining (a marker of apoptosis^34^) in both male and female mice (Table 1). Staining in the negative controls and the 0.1 mg/kg GenX groups was indistinguishable in both males and females. Representative staining in the male control group is shown in Figure 1A and B. In male mice exposed to 0.5 mg/kg group, caspase-3 immunoreactivity was observed in the cytoplasm in cells with normal histology (Figure 1C and D). Only 1 of 5 female mice exhibited such staining at 0.5 mg/kg (Table 1). In the 5 mg/kg group, caspase-3 staining was present in the cytoplasm of many cells, the nucleus of some cells, and in apoptotic figures (Figure 1E and F). Three of the 5 males exhibited scoring grade 4, whereas only 1 female exhibited a grade 4 (see Supplemental Table S2 for the number of animals with various grades of caspase-3 staining). Broadly, the cytoplasmic staining of intact hepatocytes tended to occur proximal to the central veins (Figure 1C and E).

### Transcriptomic Changes Associated With Exposure

Data from a single sample were removed from the analysis according to the sequencing depth criterion detailed above, resulting in a total of 39 samples analyzed for gene expression. The removed sample was from a female in the 0.5 mg/kg group. The expression levels of 21,448 mouse genes, as measured by 35,121 probes (Supplemental Table S3), were reported from the TempO-seq experiment. The total number of genes with significantly altered expression levels across the treatment groups ranged from 62 (females administered 0.1 mg/kg) to 1406 (males administered 5 mg/kg; Table 2 and Supplemental Table S3). For all exposure groups, there was a fairly even split between DEGs with increased expression compared to controls and those with decreased expression (Table 2).

**Table 2.** Number of Differentially Expressed Genes for Each Treatment Group.

Sex	Direction	GenX Treatment (mg/kg)		
		0.1	0.5	5
Male	Up	94	354	749
	Down	46	232	657
	Total	140	586	1406
Female	Up	22	83	384
	Down	40	69	327
	Total	62	142	711

### Gene Set Enrichment Analysis Reveals Dose-Responsive Changes and Peroxisomal Signaling

The results from the 2 methods employed to identify enrichment of biological and molecular gene sets and pathways (GSEA preranked list vs. hypergeometric test, see section “Materials and Methods”) were similar insofar as the most highly significantly enriched gene sets for each treatment group were similar according to either method (Supplemental Tables S4 and S5). Using a total of 1329 gene sets from the CP subcollection of the Curated Gene Sets collection, there were no gene sets with enrichment at the 0.1 mg/kg bw/d concentration for either sex. At the 0.5 mg/kg concentration, according to the GSEA preranked method, there were 65 enriched gene sets for females and 31 for males, using an FDR <10% as a cutoff value for significant enrichment (Supplemental Table S4). At 5 mg/kg, there were 154 enriched gene sets for females and 125 for males. Generally, any gene set that was enriched in both the 0.5 and the 5 mg/kg groups was more significantly enriched in the higher concentration group, due to a greater number of the genes within those gene sets being significantly differentially expressed at the higher concentration.

#### Gene set enrichment analysis reveals significant changes to peroxisome-related signaling

Gene sets related to fatty acid metabolism, xenobiotic metabolism, peroxisome processes, and adipogenesis were among the topmost enriched gene sets in samples from the 0.5 and 5 mg/kg dose groups for both sexes (Table 3).

**Table 3.** Top 10 Most Significantly Enriched Mouse Liver Gene Sets for Each Treatment Group Using the GSEA Method.

GenX Treatment (mg/kg)	Gene Set Name	Adjusted *P* Value	Overall Direction
Males			
0.1	None	NA	NA
0.5	KEGG PPAR signaling pathway	.0001	Up
	KEGG fatty acid metabolism	.0001	Up
	KEGG peroxisome	.00017	Up
	KEGG valine leucine and isoleucine degradation	.00022	Up
	KEGG lysine degradation	.00265	Up
	REACTOME mitochondrial fatty acid beta oxidation	.00293	Up
	REACTOME peroxisomal lipid metabolism	.00704	Up
	REACTOME TCA cycle and respiratory electron transport	.00750	Up
	PID HNF3A pathway	.00783	Up
	KEGG complement and coagulation cascades	.0101	Down
5	KEGG PPAR signaling pathway	<.0001	Up
	KEGG fatty acid metabolism	<.0001	Up
	KEGG nitrogen metabolism	<.0001	Up
	REACTOME complement cascade	<.0001	Down
	REACTOME formation of fibrin clot clotting cascade	<.0001	Down
	KEGG complement and coagulation cascades	<.0001	Down
	NABA ECM regulators	.00111	Down
	BIOCARTA comp pathway	.00134	Down
	KEGG glycine serine and threonine metabolism	.00146	Down
	REACTOME metabolism of amino acids and derivatives	.00164	Down
Females			
0.1	None	NA	NA
0.5	PID UPA UPAR pathway	<.0001	Down
	KEGG complement and coagulation cascades	<.0001	Down
	KEGG fatty acid metabolism	<.0001	Up
	KEGG valine leucine and isoleucine degradation	<.0001	Up
	KEGG peroxisome	<.0001	Up
	KEGG PPAR signaling pathway	<.0001	Up
	KEGG butanoate metabolism	.00010	Up
	REACTOME cholesterol biosynthesis	.00190	Up
	REACTOME mitochondrial fatty acid beta oxidation	.00219	Up
	KEGG steroid biosynthesis	.00328	Up
5	REACTOME metabolism of mRNA	<.0001	Up
	KEGG Huntington disease	<.0001	Up
	REACTOME fatty acid triacylglycerol and ketone body metabolism	<.0001	Up
	KEGG oxidative phosphorylation	<.0001	Up
	KEGG Parkinson disease	<.0001	Up
	REACTOME TCA cycle and respiratory electron transport	<.0001	Up
	REACTOME CDT1 association with the CDC6 ORC origin complex	<.0001	Up
	REACTOME peptide chain elongation	<.0001	Up
	KEGG peroxisome	<.0001	Up
	REACTOME nonsense mediated decay enhanced by the exon junction complex	<.0001	Up

Abbreviations: CD6:ORC, cell division cycle 6: origin recognition complex; CDT1, Chromatin licensing and DNA replication factor 1; ECM, extracellular matrix; HNF3A, hepatocyte nuclear factor 3-α; KEGG, Kyoto Encyclopedia of Genes and Genomes; mRNA, messenger RNA; NA, not applicable; PID, Pathway Interaction Database; PPAR, peroxisome proliferator-activated receptor; REACTOME, Reactome database of reactions, pathways, and biological processes; TCA, tricarboxylic acid; UPA, urokinase-type plasminogen activator; UPAR, urokinase-type plasminogen activator and its receptor.

**Figure 2.**
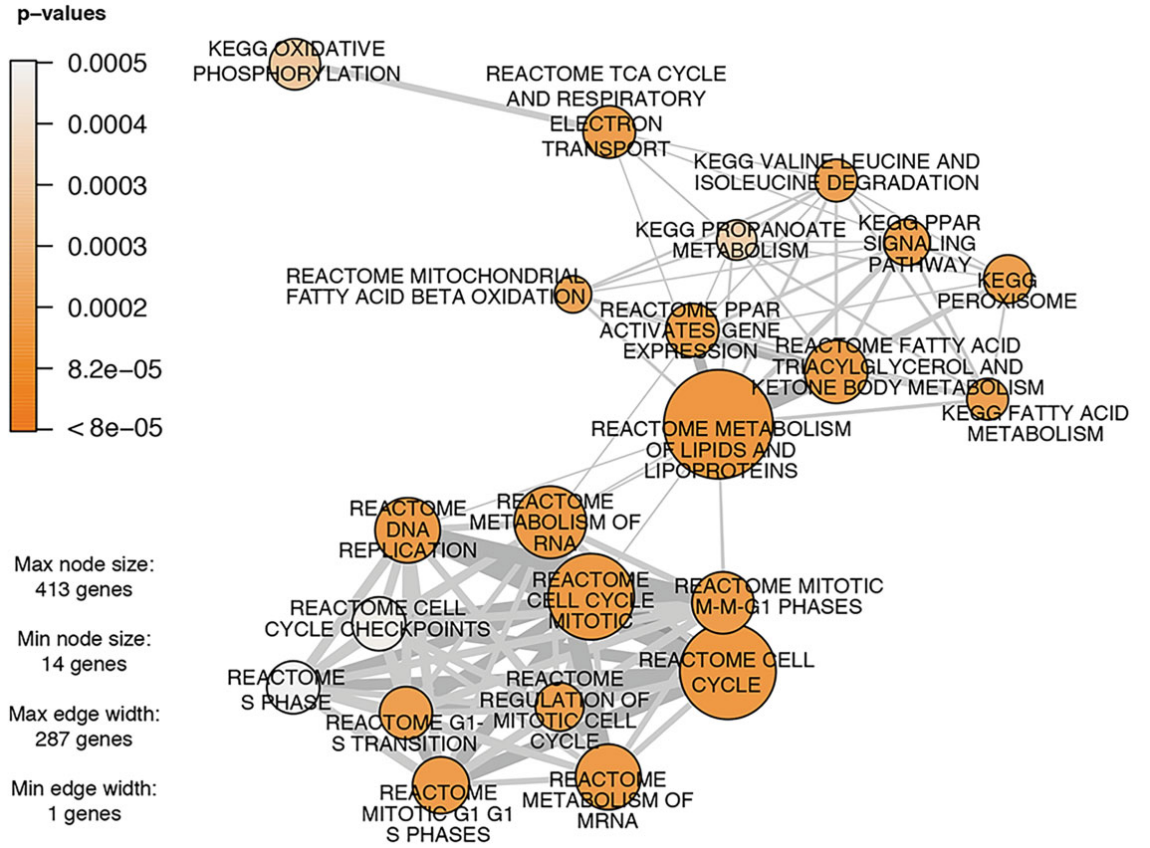
Network plots for gene set enrichment analysis. Network plot showing enriched upregulated canonical pathways for gene expression data from male mice exposed to 5 mg GenX/kg bw/d. For each pathway, significance is represented by the color shading scale of the nodes according to *P* value, and the number of genes in each pathway is represented by node size. Nodes are connected by lines that represent individual genes in the data set which are common to multiple nodes. The thickness of these connector lines represents the number of common genes. A *P* value of 5 × 10E-4 was used as a cutoff for the gene sets visualized to represent the topmost enriched gene sets.

**Figure 3.**
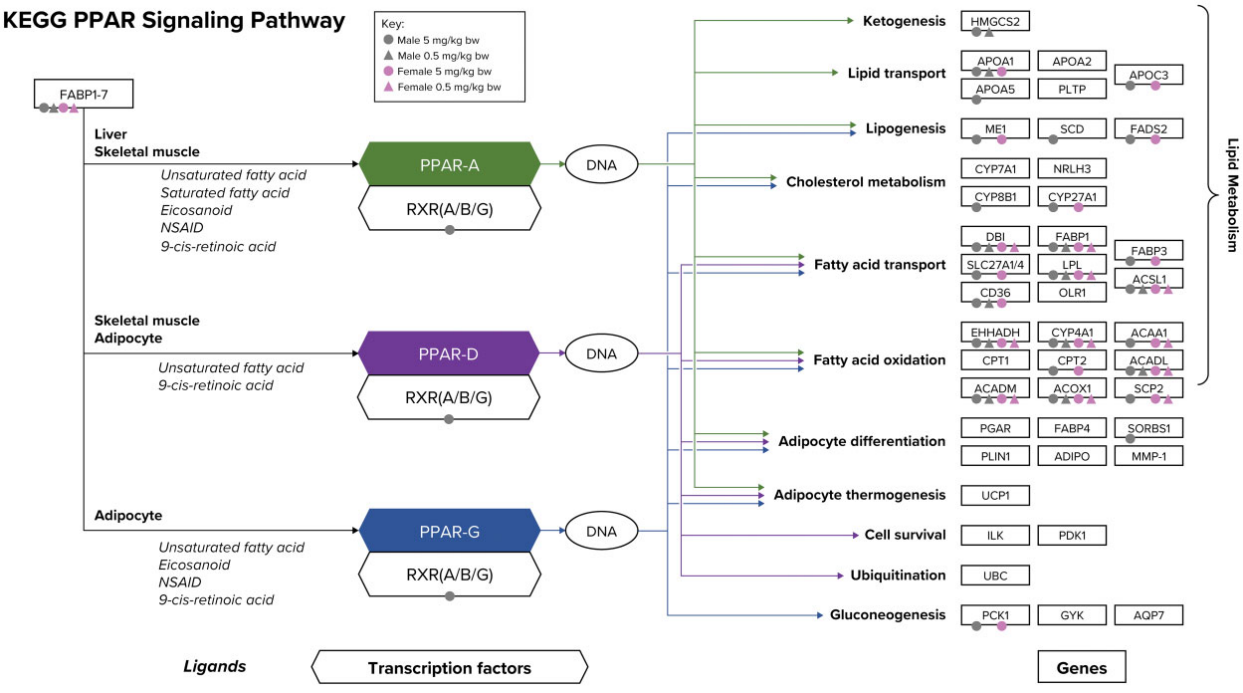
The PPAR signaling network. Ligands, transcription factors, and genes as related to PPAR α/δ/γ signaling are shown according to the KEGG database. Individual genes that are significantly differentially expressed in the present study are notated by color-coded shapes according to sex (males = gray, females = pink) and concentration (5 mg/kg bw/d = circles, 0.5 mg/kg bw/d = triangles). Arrows corresponding to each PPAR family member show the target genes for that family member (α/δ/γ): green = PPARα, purple = PPARδ, and blue = PPARγ. Bw indicates body weight; KEGG, Kyoto Encyclopedia of Genes and Genomes; PPAR, peroxisome proliferator-activated receptor.

Multiple gene sets related to peroxisome proliferator-activated receptor (PPAR) signaling, general peroxisome-related genes, or fatty acid metabolism include many of the same genes. Thus, many of the same DEGs included in these gene sets are responsible for the enrichment of these pathways (Figure 2, Supplemental Table S4). The genes that are included in the KEGG gene set “PPAR Signaling Pathway” that were altered by GenX exposure at 0.5 and 5 mg/kg bw/d in both sexes are acetyl-CoA acyltransferase 1 (*Acaa1*), acyl-CoA dehydrogenase medium chain (*Acadl*), acyl-CoA dehydrogenase long chain (*Acadm*), acyl-CoA oxidase 1 (*Acox1*), acyl-CoA synthetase long-chain family member 1 (*Acsl1*), cytochrome P450 family 4 subfamily A member 22 (*Cyp4a22*), diazepam-binding inhibitor, acyl-CoA binding protein (*Dbi*), enoyl-CoA hydratase and 3-hydroxyacyl-CoA dehydrogenase (*Ehhadh*), fatty acid binding protein 1 (*Fabp1*), and sterol carrier protein 2 (*Scp2*) (Figure 3, Supplemental Table S4). Additional isoforms from the same families of which these genes are members were significantly differentially expressed at the 5 mg/kg bw/d concentration. The apolipoprotein family members *Apoa1*, *Apoa5*, and *Apoc3*, CD36 molecule (*Cd36*, also known as fatty acid translocase), fatty acid desaturase 2 (*Fads2*), malic enzyme 1 (*Me1*), phosphoenolpyruvate carboxykinase 1 (*Pck1*), and solute carriers (*Slc27a1*, *Slc27a4*, *Slc27a5*), all of which are also in the KEGG “PPAR Signaling Pathway”, were differentially expressed at the 5 mg/kg bw/d concentration in both sexes. Retinoid X receptor α (*Rxra*) was uniquely upregulated in males at the 5 mg/kg bw/d group. Many of these genes are also present in related gene sets that were also significantly enriched, including “KEGG Fatty Acid Metabolism,” “KEGG Peroxisome,” “REACTOME Peroxisomal Lipid Metabolism,” “BIOCARTA PPARA Pathway,” and “REACTOME PPARA Activates Gene Expression,” among others. Gene sets that are specific to PPARα, as compared to the more general PPAR signaling gene sets, were significantly enriched at the highest concentration for both sexes, and in males at the 0.5 mg/kg bw/d concentration (Supplemental Table S4).

Regarding a signal specific to the PPARα subtype of the receptor family, an investigation of the various genes included in the signaling pathways for PPARα, PPARδ, and PPARγ, specificity for PPARα is apparent based on the differential expression of genes that are specific to the α subtype and the lack of changes in expression of most of the genes that are specific to other subtypes (Figure 3). Notably, the *Pck1* gene was downregulated by GenX, whereas it would be expected to be upregulated upon activation of PPARγ, as a PPARγ-binding site is in the promoter region of the gene.^35^

Individual genes in the acyl-CoA oxidase family, which are common to the peroxisomal signaling pathways, appear to be the underlying cause of enrichment for additional disease-related gene sets, such as those related to Parkinson disease, Huntington disease, Alzheimer disease, and with oxidative phosphorylation. NADH:ubiquinone oxidoreductase subunit coding genes are also responsible for the enrichment of such gene sets in this data set. The enrichment of these gene sets is unique to female mice, with the exception of oxidative phosphorylation in males only at the highest concentration (Supplemental Table S4).

#### Gene set enrichment analysis reveals significant changes to cellular processes and cell cycle mediators, apoptosis

Although the signal was not as strong as the signal for PPAR and fatty acid metabolism signaling, significant enrichment of various cell cycle and messenger RNA (mRNA) processing gene sets, such as “REACTOME Regulation of Apoptosis” and “REACTOME Regulation of Mitotic Cell Cycle,” was observed. These cell cycle–related pathways were only enriched at the 5 mg/kg concentration, in both sexes, and the enrichment was primarily driven by the upregulation of genes that encode for proteasome subunits. The enrichment of various cellular process gene sets driven by proteasome subunits was evident to a much greater degree in the female mice compared to the males, particularly at the 5 mg/kg concentration. Relatedly, “REACTOME p53-Independent G1-S DNA Damage Checkpoint” was also enriched at 5 mg/kg for both sexes, and the “REACTOME p53-Independent G1-S DNA Damage Checkpoint” gene set was enriched for females only at 5 mg/kg bw/d. Importantly, all of the genes driving the enrichment of this gene set were proteasome subunits and ribosomal proteins, while the *Chek1* and *Chek2* checkpoint genes were not significantly altered, nor was the ataxia-telangiectasia mutated (*Atm*) gene. Notably, no other DNA damage-related gene sets were enriched in either sex at any dose.

#### Gene set enrichment analysis reveals downregulation of coagulation factor expression

Several gene sets related to coagulation and clotting cascade were enriched among downregulated genes, for both sexes and at both the 0.5 and 5 mg/kg doses, such as “KEGG Complement and Coagulation Cascades,” “REACTOME Complement Cascade,” and “REACTOME Formation of Fibrin Clot Clotting Cascade.”

#### Confirmation of GSEA ranked-list results using hypergeometric test

As a means to confirm the results obtained using the GSEA ranked list with all gene expression information, a hypergeometric test was conducted to evaluate enrichment using only genes that met statistical criteria for significant differential expression (FDR <10%). This test does not take into account the magnitude of change (ie, fold-change of the individual genes) and can be run irrespective of direction of change. To understand if the same gene sets were significantly enriched when only the most significantly altered genes were used in the enrichment analysis, we conducted the hypergeometric test, or “overrepresentation analysis,” as a secondary analysis. A clear dose–response was observed in the gene set enrichment using this method, similar to the GSEA ranked method. Further, gene sets related to peroxisome signaling and fatty acid metabolism were the most significantly enriched for both sexes, in a dose-responsive manner. At the 0.5 mg/kg concentration, there were only 18 enriched gene sets for females and 80 for males, using an FDR <10% as a cutoff value for significant enrichment and including genes altered in both directions in the test set. At 5 mg/kg, there were 152 enriched gene sets for females and 181 for males (Supplemental Table S5). Similar to the GSEA method, gene sets that were enriched in both the 0.5 and the 5 mg/kg groups were more significantly enriched in the higher concentration group, due to a greater number of the genes within those gene sets being significantly differentially expressed at the higher concentration. Importantly, PPAR-related gene sets were highly significantly upregulated and also represent the overwhelming majority of the topmost significantly enriched gene sets (Table 4). The gene set with the most significant enrichment across all treatment groups (ie, the smallest adjusted *P* value) was “KEGG PPAR Signaling Pathway” for male mice in the 5 mg/kg group.

**Table 4.** Top 10 Most Significantly Enriched Liver Gene Sets for Each Treatment Group Using the Hypergeometric Test.

GenX Treatment (mg/kg)	Gene Set Name	Adjusted *P* Value	Overall Direction
Males			
0.1	None	NA	NA
0.5	KEGG fatty acid metabolism	2.61E-12	Up
	KEGG peroxisome	1.15E-09	Up
	KEGG complement and coagulation cascades	4.79E-08	Down
	REACTOME fatty acid triacylglycerol and ketone body metabolism	4.22E-07	Up
	KEGG biosynthesis of unsaturated fatty acids	4.34E-07	Up
	REACTOME mitochondrial fatty acid beta oxidation	4.34E-07	Up
	KEGG valine leucine and isoleucine degradation	1.24E-05	Up
	KEGG lysine degradation	1.91E-05	Up
	KEGG PPAR signaling pathway	1.91E-05	Up
	REACTOME alpha linolenic acid ALA metabolism	4.47E-05	Down
5	KEGG PPAR signaling pathway	3.52E-19	Up
	REACTOME metabolism of amino acids and derivatives	5.17E-17	Down
	REACTOME metabolism of lipids and lipoproteins	1.86E-16	Up
	KEGG complement and coagulation cascades	6.00E-16	Down
	KEGG fatty acid metabolism	6.00E-16	Up
	REACTOME fatty acid triacylglycerol and ketone body metabolism	1.67E-11	Up
	KEGG peroxisome	7.12E-11	Up
	KEGG valine leucine and isoleucine degradation	8.39E-10	Up
	REACTOME 3-UTR mediated translational regulation	9.69E-10	Up
	KEGG biosynthesis of unsaturated fatty acids	3.69E-09	Up
Females			
0.1	None	NA	NA
0.5	KEGG fatty acid metabolism	4.66E-13	Up
	REACTOME alpha linolenic acid ALA metabolism	6.26E-08	Up
	KEGG PPAR signaling pathway	6.26E-08	Up
	KEGG peroxisome	1.90E-07	Up
	KEGG valine leucine and isoleucine degradation	4.78E-07	Up
	REACTOME fatty acid triacylglycerol and ketone body metabolism	1.62E-06	Up
	REACTOME mitochondrial fatty acid beta oxidation	1.05E-05	Up
	KEGG biosynthesis of unsaturated fatty acids	5.19E-05	Up
	REACTOME metabolism of lipids and lipoproteins	7.89E-05	Up
	KEGG propanoate metabolism	5.62E-04	Up
5	KEGG fatty acid metabolism	1.36E-19	Up
	REACTOME TCA cycle and respiratory electron transport	3.81E-18	Up
	REACTOME metabolism of lipids and lipoproteins	3.86E-18	Up
	KEGG PPAR signaling pathway	6.40E-18	Up
	KEGG peroxisome	2.90E-16	Up
	REACTOME respiratory electron transport ATP synthesis by chemiosmotic coupling and heat production by uncoupling proteins	5.37E-14	Up
	KEGG complement and coagulation cascades	9.76E-14	Down
	REACTOME fatty acid triacylglycerol and ketone body metabolism	6.53E-13	Up
	REACTOME respiratory electron transport	2.36E-12	Up
	REACTOME metabolism of amino acids and derivatives	4.08E-12	Up

Abbreviations: ATP, adenosine triphosphate; KEGG, Kyoto Encyclopedia of Genes and Genomes; PPAR, peroxisome proliferator-activated receptor; REACTOME, Reactome database of reactions, pathways, and biological processes; TCA, tricarboxylic acid; UTR, untranslated region.

Using the Enrichr tool to confirm the significance of PPAR signaling and/or to identify any additional signaling pathways of significance, PPAR signaling pathways (including PPARα-specific pathways), fatty acid metabolism, and complement and coagulation cascade-related pathways were found to be the top 10 most significantly enriched pathways according to gene sets available from platforms other than those included in our primary analysis using the MSigDB gene sets, such as curated gene sets available from NCATS BioPlanet^36^ and WikiPathways^37^ (Supplemental Table S6). The results for the enrichment of gene sets for the KEGG, Reactome, and BioCarta databases according to the analysis presented above were also confirmed using the Enrichr software (data not shown).

### Benchmark Dose Modeling

**Figure 4.**
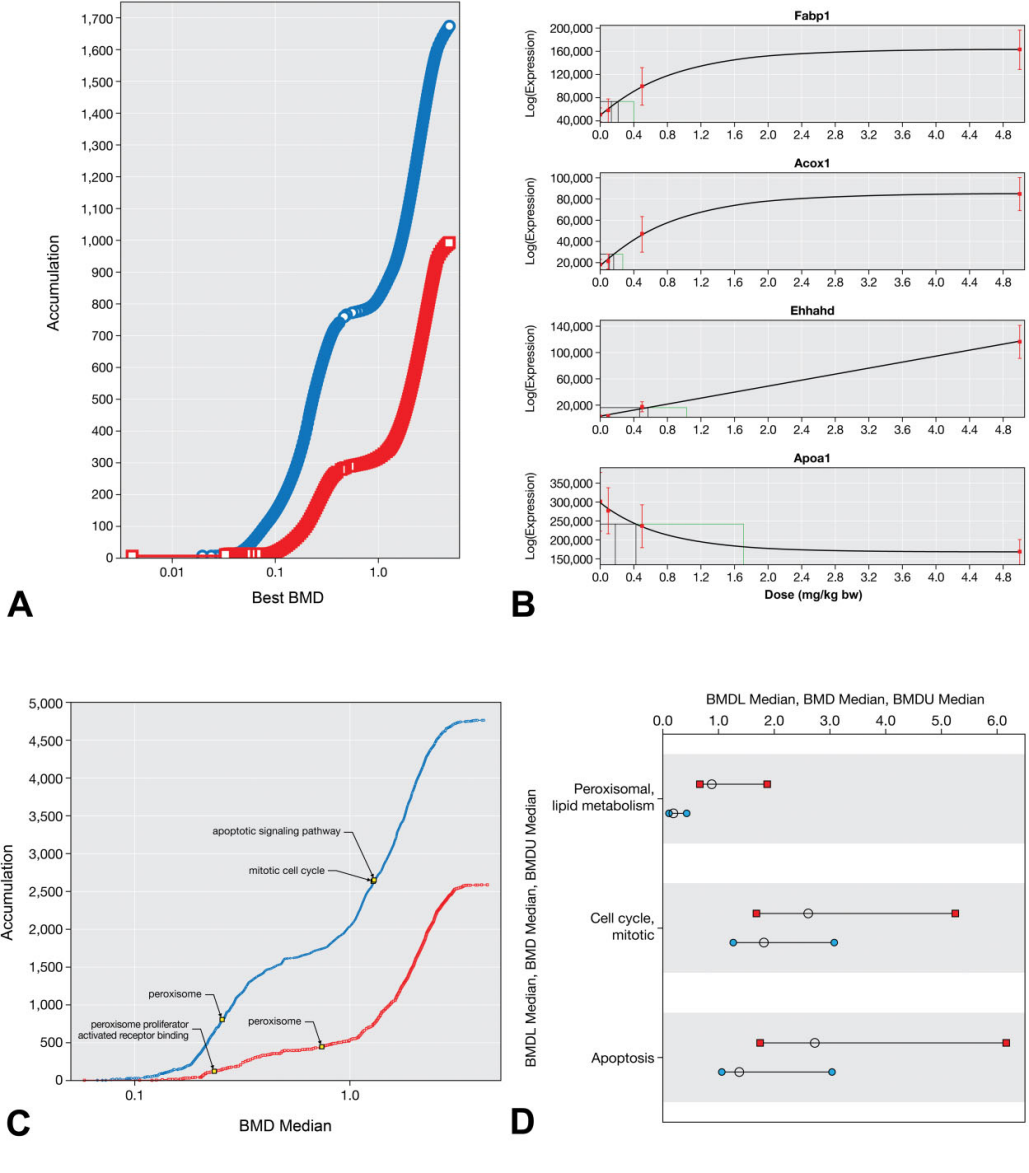
BMD analysis. A, Accumulation plots for best BMD values for DEGs in male (blue) and female (red) mice. B, Example BMD model fits for PPAR-related genes *Fabp1*, *Acox1*, *Ehhahd*, and *Apoa1* in male liver. Models for each example visualization were chosen based on goodness of fit and biological plausibility. Full results including BMDs for all models for each gene are included in Supplemental Table S7. Red squares and whiskers represent mean and standard deviation, respectively, across all samples in the dose group. The drop-down lines in each plot represent the BMDL, BMD, and BMDU values. C, Accumulation plots for the BMD values for GO terms in male (blue) and female (red) mice. D, Range plots for the BMD, BMDL, and BMDU for select REACTOME pathways in male (blue) and female (red) mice. Selected terms in C and D are phenotypically linked to observed liver pathology. BMD indicates benchmark dose, BMDL, benchmark dose (lower confidence limit); BMDU, benchmark dose (upper confidence limit); DEG, differentially expressed gene; GO, Gene Ontology; PPAR, peroxisome proliferator-activated receptor; REACTOME, Reactome database of reactions, pathways, and biological processes.

The dose–response for individual genes and signaling pathways were analyzed and visualized using BMD modeling. Consistent with the increased incidence of liver effects in males relative to females, the overall transcriptomic responses in males were more sensitive than females, as evidenced by lower BMD values (Figure 4A). Functional classification using significantly dose-responsive genes indicated similar results as the gene set enrichment analysis conducted on individual treatment groups; specifically, gene sets related to peroxisomal signaling, apoptosis, and mitosis were all significantly enriched. Examples of BMD curve are shown for the PPAR-related genes *Fabp1*, *Apoa1*, *Acox1*, and *Ehhahd* (Figure 4B). The pathway-level median BMD values for GO terms and REACTOME pathways were overall lower in male mice than female mice (Figure 4C and D). As shown on the accumulation plots and the range plots (Figure 4C and D), the median BMD values for PPAR-related pathways are much lower than for those related to apoptosis and mitosis. These transcriptomic results are consistent with evidence for increased peroxisomal activity (eg, hepatocellular hypertrophy, acyl-CoA enzyme activity) in the lower dose groups and apoptosis and mitosis in high-dose group. The full results from the BMD analysis and the functional classification conducted using BMD modeling can be found in Supplemental Tables S7 and S8, respectively.

## Discussion

Perfluoroalkyl and polyfluoroalkyl substance compounds are commonly used in industrial and consumer products and are persistent in the environment.^1^ The biopersistence and long half-lives of PFAS in blood have raised concerns regarding the safety of exposure to this group of compounds. However, the results of the present study indicate that GenX induces liver toxicity in mice via a mechanism involving PPARα.

The dose-dependent increases in liver hypertrophy and liver organ weight previously reported^8^ align with known features of PPARα-induced liver cancer in rodents.^38,39^ The reevaluation of single-cell necrosis as apoptosis using updated diagnostic criteria described by Elmore et al^10^ was confirmed by caspase-3 staining. Further, an increase in mitotic bodies was also observed in male mice at the same concentration as the significant increase in apoptosis (the highest exposure group). These findings align with a similar reevaluation of male mice exposed to GenX for approximately 90 days.^6,8^ Therein, it was argued that apoptosis was part of a PPARα MOA and not indicative of liver toxicity per se. According to the Hall criteria^40^ for assessing liver toxicity, sequelae related to PPARα have limited human relevance. As such, Thompson et al^6^ concluded that apoptosis should not serve as the basis for a human health risk assessment. Coupled with transcriptomic results (see below), the present study further supports involvement of a PPARα MOA in GenX-induced liver changes.

Transcriptional changes are understood to be a primary response to chemical exposure that precede toxicity; thus, collecting gene expression data provides insight into the molecular underpinnings of toxicity.^14^ However, evaluating the whole transcriptome in an experiment offers both a wealth of knowledge and a challenge in inherent complexity. It has been put forth that a major challenge in the application of transcriptomic data to human health risk assessments is gaining an understanding of how to best evaluate complex gene expression data into understandable and applicable information.^14^ Although changes to individual genes can provide information regarding dose–response relationships and no- or low-effect levels for transcriptional changes, querying the entire transcriptome or a large subset of genes that represent a broad range of biological pathways provides information regarding alterations to signaling networks that carry biological relevance. Such changes at the signaling pathway level play important roles in higher order biological processes that are important components of pathological changes and disease states. Using two different methods for gene set enrichment analysis, we were able to test which gene sets are most closely correlated with treatment based on a ranking approach for all genes (the GSEA method), and we also confirmed those results by applying a filter for only significantly DEGs without any ranking and conducting an overrepresentation analysis (hypergeometric test). In both cases, PPAR signaling and related pathways were among the most highly significantly enriched gene sets, providing clear evidence that GenX induces PPAR. Although the underlying mechanism appears to be the same in both sexes, male mice present higher sensitivity to the GenX-induced transcriptomic alterations than females, as evidenced by the overall higher number of altered genes (Table 2) and the lower BMDs for several signaling pathways relevant to the observed histopathological changes (Figure 4D). This aligns with the higher sensitivity of males to liver toxicity at the phenotypic level as evaluated by histopathology in the same experiment.^8^ The underlying reason for the sex difference at the dose level is unknown; although variation in toxicokinetics may be relevant, minimal differences in the toxicokinetics of GenX between sexes have been reported.^5^ This transcriptional signal provides key evidence in the understanding of the MOA of GenX and mouse liver toxicity. The enrichment of these gene sets was accompanied by enrichment of cell cycle regulators and mitotic signaling, demonstrating the proliferative response occurring in tandem (or resultant of) the upregulation of PPAR signaling. Further, transactivation assays conducted in cell lines overexpressing mouse or rat PPARα demonstrated that GenX activated both mouse and rat PPARα, albeit with different potencies (Supplemental Figure S2).

Recently, GenX was shown to induce transcriptomic changes in rat dams exposed to GenX by oral gavage on gestational days 14 to 18, as well as in fetal liver.^41^ Maternal livers displayed upregulation of genes involved in PPAR signaling pathways, including fatty acid metabolism and cell proliferation. *Ehhadh*, coding for the peroxisomal enzyme 3-hydroxyacyl CoA dehydrogenase, was the most highly upregulated gene, increasing 3-fold at 3 mg/kg/d and 55-fold at 500 mg/kg/d. In fetal livers, there was significant upregulation of genes in the PPAR signaling pathway and they were associated with fatty acid metabolism, but not cell proliferation. Several genes were upregulated in fetal liver beginning at 3 mg/kg/d; most notably, *Ehhadh* was upregulated 5-fold at 3 mg/kg/d and over 300-fold at 500 mg/kg/d. Although the induction of PPAR signaling in the fetal liver following maternal exposure to GenX is clear, the significance of these transcriptomic responses is unknown. A recent 2-year bioassay with a PPARα activator (PFOA) reported no difference in tumorigenic responses between rats first exposed to PFOA in utero versus those first exposed after weaning.^42^ Studies in PPARα-null mice and those expressing human PPARα have been shown to be refractory to developmental effects elicited by PFOA in wild-type mice.^43^ Overall, the human relevance of fetal channels in PPARα signaling is uncertain. Notably, the transcript changes in rat livers at 3 mg/kg/d GenX is equivalent to approximately1 mg/kg/d in humans based on allometric scaling. Importantly, we recently proposed a reference dose of 0.01 mg/kg/d, a value that is 100-fold lower than our estimated human equivalent dose based on the observed transcript changes.^6^

Another proposed concurrent, although not “key,” event in the MOA for PPARα-dependent rodent liver cancer is an increase in nuclear factor-κB.^38^ In the present study, the gene set “REACTOME Activation of NF KappaB in B cells” was significantly enriched in the upregulated direction for both males and females exposed to 5 mg/kg bw/d (Supplemental Tables S4 and S5).

While the signal in this study provides clear evidence of alterations to peroxisomal signaling, other transcriptional targets and/or mechanisms related to liver toxicity were also investigated. Whole transcriptome sequencing enables the ability to investigate alterations to a broad range of signaling pathways, rather than the limitation of only querying specific pathways by measuring related genes. In fact, the gene expression data may be analyzed for the involvement of as many gene sets or pathways as are well characterized. Downregulation of coagulation cascade proteins during hyperplasia-mediated liver regeneration in mice has been previously demonstrated,^44^ similar to the downregulation in coagulation cascade mRNA signaling observed in the present study. Because of previous reports of GenX-induced steatosis,^11^ this phenotype and underlying molecular signaling were also reviewed. Steatosis was unremarkable by histological evaluation of H&E-stained liver sections across treatment and control groups. The sterol regulatory element binding transcription factors 1 and 2 (*Srebf1* and *Srebf2*) genes that are involved in fatty acid and cholesterol synthesis were unchanged in all treatment groups, as was the SREBF chaperone (*Scad*) gene (Supplemental Table S3), relative to controls. Further, when compared to a list of 99 genes that have been recently characterized as a biomarker for SREBP activation based on concordance of DEGs in transgenic SREBP-overexpressing mice and further tested for predictability using various other PFAS that cause steatosis,^45^ the overlap with DEGs in the 5 mg/kg dose group in the present study was only 19% and 30% for females and males, respectively.

Other perfluoroalkyl substances, specifically perfluoroalkyl acids, have been shown to activate other receptors, such as the constitutive androstane receptor (CAR) and estrogen receptor α^46,47^; however, no such signal was evident for GenX in the liver. For example, the direct targets of CAR *Cyp2b10* and *Cyp2c55* were not altered by GenX, and only 13 of the 83 genes that have been designated as a CAR biomarker signature^48^ were altered by GenX in the predicted direction by the signature in males at 5 mg/kg. Transcriptomic signaling related to other nuclear receptors, which were investigated to further inform the MOA underlying GenX-induced liver toxicity, was not significantly altered by GenX. For example, AhR signature genes *SLC10A1* and *SLCO1B1*^49^ were not significantly induced in any treatment group (Supplemental Table S3). In fact, *Slc10a1* was significantly reduced in the female 5 mg/kg group and male 0.5 and 5 mg/kg groups, and *Slco1b2* (the mouse homolog of *SLCO1B1*) was reduced in the male 5 mg/kg group. Gene sets specific to androgen and estrogen receptor signaling were unchanged in all treatment groups (Supplemental Tables S4 and S5).

It is well accepted that species differences in PPARα expression and activity exist, and it has been proposed that Syrian hamsters, guinea pigs, and nonhuman primates are better representative model organisms for human-relevant responses to PPARα activation compared to mice and rats.^50^ Although the species considered to be more human relevant (hamsters, guinea pigs, and primates) exhibit alterations to genes and proteins involved in lipid homeostasis that represent the underlying hypolipidemic effects of PPARα activation, important key events downstream of PPARα activation do not occur, including alteration of cell growth pathways, hepatocyte proliferation, and liver tumors. Further, minimal or no effects have been observed on cell growth pathways and hepatocellular proliferation in human primary hepatocytes exposed to PPARα agonists.^38,51^ Species-specific differences also exist at the individual gene level in the signaling pathway. For example, while PPARα activation increases plasma levels and hepatic mRNA expression of apolipoprotein A 1 (*APOA1*) in humans, the opposite is observed in rodents (reduced *Apoa1*)^52^; this was evident in the present study (see Figure 4, Supplemental Tables S3-5). Accordingly, it has been stated in a comprehensive review of the evidence that the consensus that the PPARα MOA lacks human relevance is supported by an “overwhelming body of evidence” and has “almost universal acceptance.”^51^ It has been demonstrated that PPARα knockout mice, as well as PPARα humanized (PPARα knockout/knock-in) mice, do not develop hepatocellular tumors in response to long-term treatment with peroxisome proliferators,^53-55^ suggesting that the human receptor is structurally and/or functionally different from the murine receptor. Because induction of cell proliferation is considered the mechanistic basis for the peroxisome proliferator-induced liver toxicity and tumorigenicity, it is likely that humans are not susceptible to such sequelae.^40^ The evidence of the lack of human relevance of PPARα MOA and liver tumors, together with the evidence that GenX acts through a PPARα MOA to induce mouse liver toxicity, has important implications in the human cancer hazard and risk assessment of GenX.

## Conclusions

An important strength of this study is the fact that the same liver tissues were used for both histopathological examination and transcriptomic analysis. This offers the ability to phenotypically anchor molecular and cellular results, affording a high level of confidence in the biological plausibility of the key events that occur following exposure. Overall, the results of the analyses presented herein provide additional evidence that liver lesions observed in mice exposed to GenX are related to PPARα and are similar to those induced by peroxisome proliferators. The results of transcriptomic analysis indicate that GenX exposure at 0.5 and 5 mg/kg bw/d for 90 days induces PPARα signaling in the liver in mice. Activation of PPARα was confirmed by a luciferase report binding assay for both mouse and rat PPARα. Together, these results support the hypothesis that GenX induces liver toxicity via a mechanism that involves activation of PPARα, which has important implications in integrating and extrapolating data from experimental rodent studies to human risk assessment due to the species specificity of PPARα activators and tumor induction; PPARα-mediated effects on cell cycle are specific to rodents.^38,39,50,51^ As such, it appears that GenX operates through a PPARα mechanism indicating species specificity, an important consideration in risk evaluations or hazard assessments of the compound.

## Supplemental Material

Supplemental Material, chappell_supplemental_fig1 - Assessment of the Mode of Action Underlying the Effects of GenX in Mouse Liver and Implications for Assessing Human Health Risks Click here for additional data file. Supplemental Material, chappell_supplemental_fig1 for Assessment of the Mode of Action Underlying the Effects of GenX in Mouse Liver and Implications for Assessing Human Health Risks by Grace A. Chappell, Chad M. Thompson, Jeffrey C. Wolf, John M. Cullen, James E. Klaunig and Laurie C. Haws in Toxicologic Pathology

Supplemental Material, Supplemental_Figure_Legends - Assessment of the Mode of Action Underlying the Effects of GenX in Mouse Liver and Implications for Assessing Human Health Risks Click here for additional data file. Supplemental Material, Supplemental_Figure_Legends for Assessment of the Mode of Action Underlying the Effects of GenX in Mouse Liver and Implications for Assessing Human Health Risks by Grace A. Chappell, Chad M. Thompson, Jeffrey C. Wolf, John M. Cullen, James E. Klaunig and Laurie C. Haws in Toxicologic Pathology

Supplemental_Materials - Assessment of the Mode of Action Underlying the Effects of GenX in Mouse Liver and Implications for Assessing Human Health Risks Click here for additional data file. Supplemental_Materials for Assessment of the Mode of Action Underlying the Effects of GenX in Mouse Liver and Implications for Assessing Human Health Risks by Grace A. Chappell, Chad M. Thompson, Jeffrey C. Wolf, John M. Cullen, James E. Klaunig and Laurie C. Haws in Toxicologic Pathology
