# Supplementary figures and images for: Assessment of the Mode of Action Underlying the Effects of GenX in Mouse Liver and Implications for Assessing Human Health Risks
Source: Toxicol Pathol. 2020 Mar 6;48(3):494–508. doi: 10.1177/0192623320905803 (PMC7153225; doi:10.1177/0192623320905803)

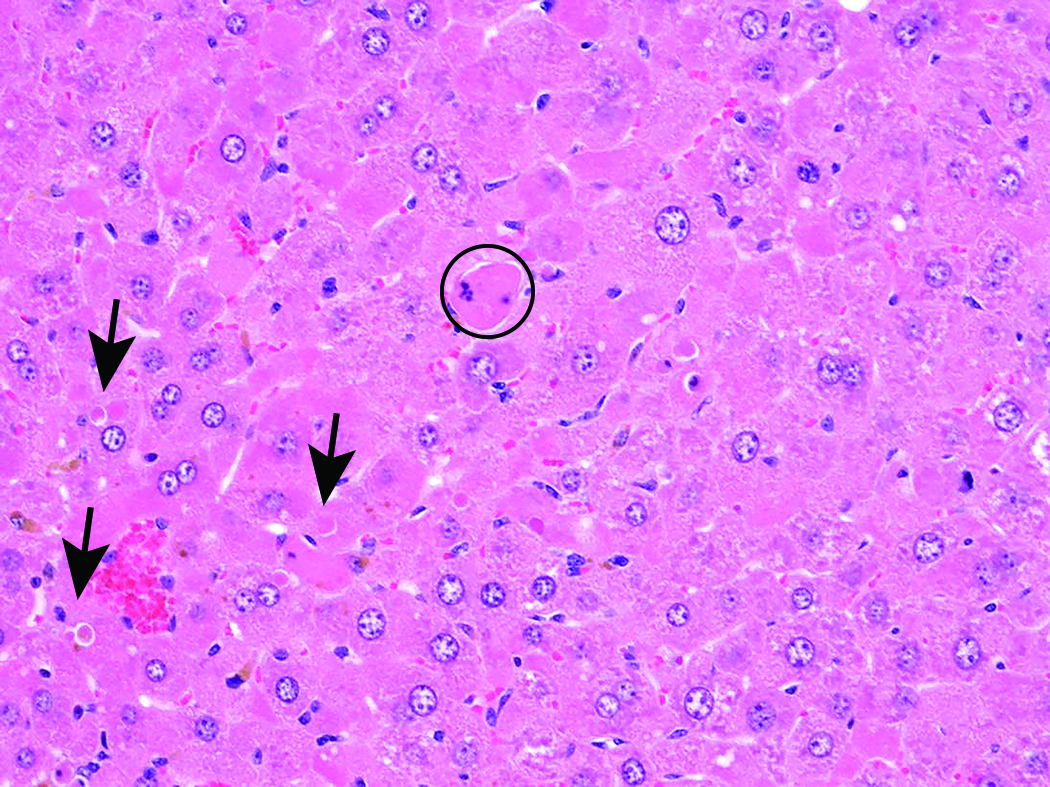

Supplement: Supplemental Material, chappell_supplemental_fig1 - Assessment of the Mode of Action Underlying the Effects of GenX in Mouse Liver and Implications for Assessing Human Health Risks [file chappell_supplemental_fig1.tif]
