# Supplementary material for: Assessment of the Mode of Action Underlying the Effects of GenX in Mouse Liver and Implications for Assessing Human Health Risks
Source: Toxicol Pathol. 2020 Mar 6;48(3):494–508. doi: 10.1177/0192623320905803 (PMC7153225; doi:10.1177/0192623320905803)
Supplement: Supplemental Material, Supplemental_Figure_Legends - Assessment of the Mode of Action Underlying the Effects of GenX in Mouse Liver and Implications for Assessing Human Health Risks [file Supplemental_Figure_Legends.docx]

Supplemental Figure Legends

Figure S1. H&E stained liver section (20x objective) from a male mouse exposed GenX for 90 days 5 mg/kg bw, exhibiting multiple apoptotic bodies (arrows) and an apoptotic hepatocyte (circled).

Figure S2. Transactivation of PPARa. Cells over-expressing mouse PPARα (A) and rat PPARα (B) were incubated with various concentrations of GenX for ~24 hours. Transactivation of PPARα was assessed by luciferase activity in reporter gene constructs. The EC50 values for the positive control GW590735 were approximately 134 nM and 917 nM for mouse and rat PPARα, respectively. The EC50 values for GenX were approximately 40 uM and 114 uM for mouse and rat PPARα, respectively.
