# Supplementary material for: Assessment of the Mode of Action Underlying the Effects of GenX in Mouse Liver and Implications for Assessing Human Health Risks
Source: Toxicol Pathol. 2020 Mar 6;48(3):494–508. doi: 10.1177/0192623320905803 (PMC7153225; doi:10.1177/0192623320905803)
Supplement: Supplemental_Materials - Assessment of the Mode of Action Underlying the Effects of GenX in Mouse Liver and Implications for Assessing Human Health Risks [file Supplemental_Materials.docx]

**Supplemental Materials**

**BMDExpress Analysis parameters**

***Benchmark Dose Analysis***

BMDExpress2 Version: BMDExpress 2.20.0180 BETA

Operating System: Windows 10

hill version: Hill Model. (Version: 2.18; Date: 03/14/2017)

power version: Power Model. (Version: 2.19; Date: 03/14/2017)

linear version: Polynomial Model. (Version: 2.21; Date: 03/14/2017)

poly 2 version: Polynomial Model. (Version: 2.21; Date: 03/14/2017)

poly 3 version: Polynomial Model. (Version: 2.21; Date: 03/14/2017)

exponential 2 version: Exponential Model. (Version: 1.11; Date: 03/14/2017)

exponential 3 version: Exponential Model. (Version: 1.11; Date: 03/14/2017)

exponential 4 version: Exponential Model. (Version: 1.11; Date: 03/14/2017)

exponential 5 version: Exponential Model. (Version: 1.11; Date: 03/14/2017)

Models fit: hill, power, linear, poly 2, poly 3, exponential 2, exponential 3, exponential 4, exponential 5

Maximum Iterations: 250

Confidence Level: 0.95

Constant Variance: 1

BMR Factor: 1.0

Restrict Power: 1

Highest Dose: 5.0

Lowest Positive Dose: 0.10000000149011612

Best Model Selection: Nested Chi Square to select best poly model followed by lowest AIC

Nested Chi Square p-value cutoff: 0.05

Fit Selected Models with Multiple Threads: 8

Number of Available Processors On Machine: 2

Destory Model Processes If Run More Than: 60000 milliseconds

Flag Hill Model with 'k' Parameter <: 1/3 of Lowest Positive Dose

Best Model Selection with Flagged Hill Model: Select Next Best Model with P-Value > 0.05

***Signaling Pathway Analyses***

Pathway Data Source: REACTOME

Organism Code: mmu

Signaling Pathway File Creation Date: 03/03/19

Deduplicate Gene Sets: false

BMDExpress2 Version: BMDExpress 2.20.0180 BETA

Remove Promiscuous Probes: true

Remove BMD > Highest Dose from Category Descriptive Statistics: true

Remove BMD with p-Value < Cutoff: 0.1

Remove genes with BMD/BMDL >: 20.0

Remove genes with BMDU/BMDL >: 40.0

Remove genes with BMD values > N fold below the lowest positive does: 10.0

Identify conflicting probe sets: 0.5

Category Count: 1301

***Gene Ontology Analyses***

GO Category: ALL

GO File Creation Date: 03/03/19

Deduplicate Gene Sets: false

BMDExpress2 Version: BMDExpress 2.20.0180 BETA

Remove Promiscuous Probes: true

Remove BMD > Highest Dose from Category Descriptive Statistics: true

Remove BMD with p-Value < Cutoff: 0.1

Remove genes with BMD/BMDL >: 20.0

Remove genes with BMDU/BMDL >: 40.0

Remove genes with BMD values > N fold below the lowest positive does: 10.0

Identify conflicting probe sets: 0.5

Category Count: 12214

**Method for *in vitro* PPARα activation assay**

PPARα activation was assessed by a transactivation assay using a luciferase reporter according to the manufacturer’s instructions (Indigo Biosciences; State College, PA). In brief, reporter cells were dispensed into the 96-well assay plate at a volume of 200 μL cell recovery medium (CRM) per well and maintained at 37°C, ≥85% humidity, 5% CO_2_. Negative controls containing only CRM were included to measure the background signal. After 4-6 hours of preincubation, the CRM was replaced with 200 μL of dosing solutions. The positive control, GW590735, was prepared in DMSO and subsequently diluted into compound screening medium (CSM). GenX was diluted directly in CSM. A positive control for cytotoxicity, staurosporine, was prepared in CSM. CMS containing 0.2% DMSO served as a vehicle control.

After 22 – 24 hours incubation, cells were washed with liver cell multiplex assay (LCMA) buffer (200 μL) prior to incubation with LCMA Reagent (50 μL) for 15 minutes at room temperature in the dark. The LCMA reagent was then replaced with Luciferase Detection Reagent (LDR) (100 μL) after which fluorescence signals (γ_ex_: 480- 490 nm; γ_em_: 525-545 nm) were recorded using a Hidex SENSE microplate reader. Signals were measured as relative fluorescence unit (RFU). All treatment conditions were run in triplicate wells. Luciferase results were plotted using Prism 8; EC_50_ values for receptor activation were estimated using the four parameter “log(agonist) vs. response” model.
